# Identification of Potential Biomarkers for Ryanodine Receptor 1 (RYR1) Mutation-Associated Myopathies Using Bioinformatics Approach

**DOI:** 10.1155/2022/8787782

**Published:** 2022-05-23

**Authors:** Xi Wang, Chang Kong, Pan Liu, Wujun Geng, Hongli Tang

**Affiliations:** ^1^Department of Anesthesia, The First Affiliated Hospital of Wenzhou Medical University, Wenzhou, Zhejiang 325000, China; ^2^Wenzhou Key Laboratory of Perioperative Medicine, Wenzhou, Zhejiang 325000, China; ^3^Department of Anesthesiology and Critical Care Medicine, Tianjin Nankai Hospital, Tianjin Medical University, Tianjin 300100, China

## Abstract

**Background:**

Myopathies related to Ryanodine receptor 1 (RYR1) mutation are the most common nondystrophy muscle disorder in humans. Early detection and diagnosis of RYR1 mutation-associated myopathies may lead to more timely treatment of patients, which contributes to the management and preparation for malignant hyperthermia. However, diagnosis of RYR1 mutation-associated myopathies is delayed and challenging. The absence of diagnostic morphological features in muscle biopsy does not rule out the possibility of pathogenic variations in RYR1. Accordingly, it is helpful to seek biomarkers to diagnose RYR1 mutation-associated myopathies.

**Methods:**

Skeletal muscle tissue microarray datasets of RYR1 mutation-associated myopathies or healthy persons were built in accordance with the gene expression synthesis (GEO) database. Differentially expressed genes (DEGs) were identified on the basis of R software. Genes specific to tissue/organ were identified through BioGPS. An enrichment analysis of DEGs was conducted in accordance with the Kyoto Encyclopedia of Genes and Genomes (KEGG) and Gene Ontology (GO). We also built protein-protein interaction (PPI) networks to explore the function and enrichment pathway of DEGs and the identification of hub genes. Lastly, the ROC curve was drawn for hub genes achieving specific expressions within skeletal muscle. Moreover, the area under the curve (AUC) was obtained to calculate the predictive value of key genes. The transcription factors of hub genes achieving specific expressions within skeletal muscle were predicted with the use of the iRegulon plugin.

**Results:**

We identified 170 DEGs among 11 muscle biopsy samples of healthy subjects and 17 muscle biopsy samples of RYR1 mutation-associated myopathy patients in the dataset. Among the above DEGs, 30 genes achieving specific expressions within tissues/organs were found. GO and KEGG enrichment analysis of DEGs mainly focused on muscle contraction, actin-mediated cell contraction, actin filament-based movement, and muscular sliding. 12 hub genes were identified with the use of Cytoscape. Four hub genes were specifically expressed in skeletal muscle tissue, including MYH1 (AUC: 0.856), TNNT3 (AUC: 0.840), MYLPF (AUC: 0.786), and ATP2A1 (AUC: 0.765). The iRegulon predicted results suggested that the transcription factor MYF6 was found with the highest reliability.

**Conclusions:**

Four skeletal muscle tissue-specific genes were identified, including MYH1, TNNT3, MYLPF, and ATP2A1, as the potential biomarkers for diagnosing and treating RYR1 mutation-associated myopathies, which provided insights into the transcriptome-level development mechanism. The transcription factor MYF6 may be a vital upstream regulator of the above biomarkers.

## 1. Introduction

Malignant hyperthermia (MH) is an autosomal dominant medicinal myopathy with a fatal hypermetabolic crisis since sarcoplasmic Ca^2+^ increases fast and uncontrollably, which arises from exposure to volatile anesthetics and/or depolarizing muscle relaxants [1–3].

Malignant hyperthermia is capable of appearing anytime in the period of early postoperative and general anesthesia (GA) [4]. The earliest symptoms of MH consist of tachycardia and increased end-expiratory carbon dioxide (ETCO_2_) levels, accompanied by muscle rigidity [4, 5]. Unrestrained skeletal muscle hypermetabolism and a sharp rise in core body temperature with changes in intracellular calcium homeostasis exacerbate cellular hypoxia, as manifested by increasing acidosis and results in the failure of vital organs [6, 7]. Without the correction of acidosis, subsequent rhabdomyolysis and muscle cell death can trigger dangerous hyperkalemia [2].

The variation of Ryanodine receptor type 1 is correlated with MH and central core disease [8, 9]. Malignant hyperthermia susceptibility (MHS) has been found as an autosomal dominant trait, largely arising from the mutation of the ryanodine receptor type 1 gene that encodes RYR1 [10, 11]. MHS, a type of dominating congenital myopathy that is inherited, is significantly correlated with central core disease (CCD). As reported by existing genetic studies conducted in numerous patients with myopathy, RYR1 variation has been most commonly reported to lead to congenital myopathy including central nuclear myopathy (CNM), congenital fibrous type disorder, and multi-micronucleus disease (MmD) [12–14]. Thus, the pathogenesis and pathologic process of RYR1 mutation-associated myopathies should be clarified and diagnosed as soon as possible. However, the potential pathogenesis of RYR1 mutation-associated myopathies remains largely unknown. In addition, the diagnosis of RYR1 mutation-associated myopathies may be challenging and requires genetic analysis, clinical manifestations, and histopathological evidence. Moreover, muscle magnetic resonance imaging (MRI) is capable of reflecting certain muscles' selective involvement [15, 16], with rare diagnostic efficiency. Accordingly, to gain insights into the pathogenesis and pathological process of malignant hyperthermia, suitable biomarkers for the diagnosis and treatment of RYR1 mutation-associated myopathies should be found.

In this study, microarray data of muscle expression profiles of RYR1 mutation-associated myopathies group and control group were obtained from GEO database, and protein-protein interaction (PPI) network investigation, functional enrichment investigation, and investigation of differential expression were used. Cytoscape identification of hub genes correlated with RYR1 mutation-associated myopathies was used to determine the core genes associated with RYR1 mutation to explore the potential pathogenesis of RYR1 mutation-associated myopathies. Four muscle-specific biomarkers were identified by BioGPS. The results of this study contribute to the explanation of the pathogenesis of RYR1 mutation-associated myopathies and are beneficial to find out the molecular mechanism of the pathological process, to provide reference for the diagnosis and therapeutic targets of RYR1 mutation-associated myopathies and help anesthesiologists to choose a reasonable anesthesia mode and prepare for malignant hyperthermia in patients.

## 2. Materials and Methods

### 2.1. Acquisition of Data of Gene Expression

Gene Expression Omnibus (GEO) has been found as the public gene expression database covering the most aspects of data, which consists of high-throughput sequencing, gene expression microarrays, and other genomic data. In this study, we employed mRNA expression profile data from 11 muscle biopsy samples from normal healthy subjects and 17 muscle biopsy samples from Ryanodine Receptor 1 (RYR1) mutation-associated myopathy patients to conduct several bioinformatics analyses. Dataset (GSE103854) originated from GEO database (https://www.ncbi.nlm.nih.gov/geo/), and the test platform referred to GPL13497.

### 2.2. Data Normalization and Identification of DEGs

We preprocessed and normalized the original files in the GEO database using the Robust Multiarray Average (RMA) in accordance with the affy package (version 1.68.0) of R software (version 4.0.1). Limma package (version 3.28.14) was employed for genetic analysis of differences between samples, and we carried out multiple testing and correction in terms of hypothesis after *p* values were conducted. False discovery rate (FDR) was adjusted to calculate the *p* value threshold, and the *p* value with the correction turned out to be *p* value adjusted [17, 18]. The standards for screening are elucidated as the adjusted *p* value < 0.05 and log2 (fold change) > 1 or <-1.

### 2.3. Heatmap and Volcano Plot Analyses

R software was adopted for creating heat and volcano maps in terms of the clearer visualization of the above DEGs. The heatmap was created based on the Pheatmap package (version 1.0.8).

### 2.4. Identify Tissue-/Organ-Specific Expressed Genes

For gaining insights into the expression specific to tissue/organ of the above DEGs, we adopted the online tool BioGPS (http://biogps.org/) for the analysis of the distribution of tissues [19]. The screening standards included the following: (1) transcripts mapped to an individual organ system characterized by expression values > 10 times the medium value; (2) no more than one-third of the second abundance tissues were expressed [20]. We considered the genes acquired in accordance with the above standards to be tissue-specific genes.

### 2.5. DEGs Analyze via GO and KEGG

The functional annotations of DEGs were assessed through the analysis of the KEGG [21–23] pathway as well as Gene Ontology (GO) enrichment [24]. DEG expression matrix was investigated through GO and KEGG enrichment to verify whether there was statistical difference between different results. Adjusted *p* value < 0.05 had statistical significance.

### 2.6. Construction of the PPI Network

We established the PPI network by the online tool STRING (https://string-db.org/) based on all DEGs on the basis of a screening condition (combined score > 0.4). Subsequently, the interactive information was acquired, and the PPI network was improved with the use of Cytoscape software (V3.8.0) in terms of more effective visualization. CytoHubba was employed for the identification of noticeable genes within the above network to be hub genes [25]. Three algorithms, including Closeness, Maximal Clique Centrality (MCC), and Degree, were adopted to obtain the top 15 hub genes [26, 27]. Lastly, for the acquisition of the final hub genes, we carried out the intersection of all relevant results.

### 2.7. ROC Analysis of Hub Genes Achieving Specific Expressions within Skeletal Muscle

The hub genes were intersected with DEGs specifically expressed in skeletal muscle tissue screened by BioGPS as candidate genes for prediction results. Draw the receiver operating characteristic (ROC) curve. We employed the pROC package (version 1.12.1) in R software for measuring the area under the curve (AUCs) and calculating the predictive value of key genes [28].

### 2.8. Transcription Factor Analysis of Hub Genes with Specific Expressions in Skeletal Muscle

iRegulon, a plugin in Cytoscape, was employed to calculate transcription factors correlated with hub genes with specific expressions within skeletal muscle [29]. The plugin used normalized enrichment score (NES) to calculate the reliability of the predicted results. The higher NES value, the higher the reliability would be. The transcription factors with NES > 5 were employed to build the regulatory network.

## 3. Results

### 3.1. Identification of DEGs

**Figure 1:**
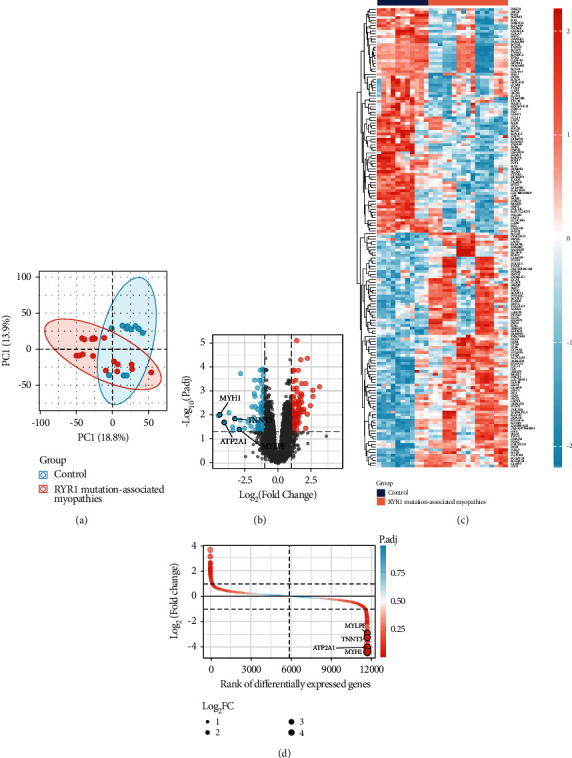
Data processing and screening of differentially expressed genes in the GSE103854 dataset. (a) PCA between RYR1 mutation-associated myopathies and normal samples. (b, c) Volcano plot and heatmap of the differentially expressed genes. (d) Rank of differentially expressed genes in GSE103854.

The mRNA expression profiles of RYR1 mutation-associated myopathies and normal skeletal muscle tissues were obtained from GEO database to determine DEGs correlated with RYR1 mutation-associated myopathies. PCA was performed to determine differences between RYR1 mutation-associated myopathies and control samples. The results suggested the differences between the two groups (Figure 1(a)). Compared with the normal group, the group of RYR1 mutation-associated myopathies was differentially expressed (|logFC| > 1, *p*.adj < 0.05). The analysis results revealed that 170 DEGs were obtained, of which 87 genes significantly increased and 83 genes significantly decreased (Figures 1(b) and 1(c)). In addition, the position of differentially expressed genes was sorted according to the differential multiple (from large to small), and the differential expression multiples are presented in Figure 1(d).

### 3.2. Identification of the Genes with Expression Specific to Tissue/Organ

With the use of BioGPS, we found 30 genes with expression specific to tissue/organ (Table 1). Among the above genes, the highest tissue/organ specific expression was observed in skeletal muscle and the pineal, and we found the second highest expression specific to the tissue/organ within adipocyte and placenta.

### 3.3. GO Enrichment and KEGG Pathway of DEGs

**Figure 2:**
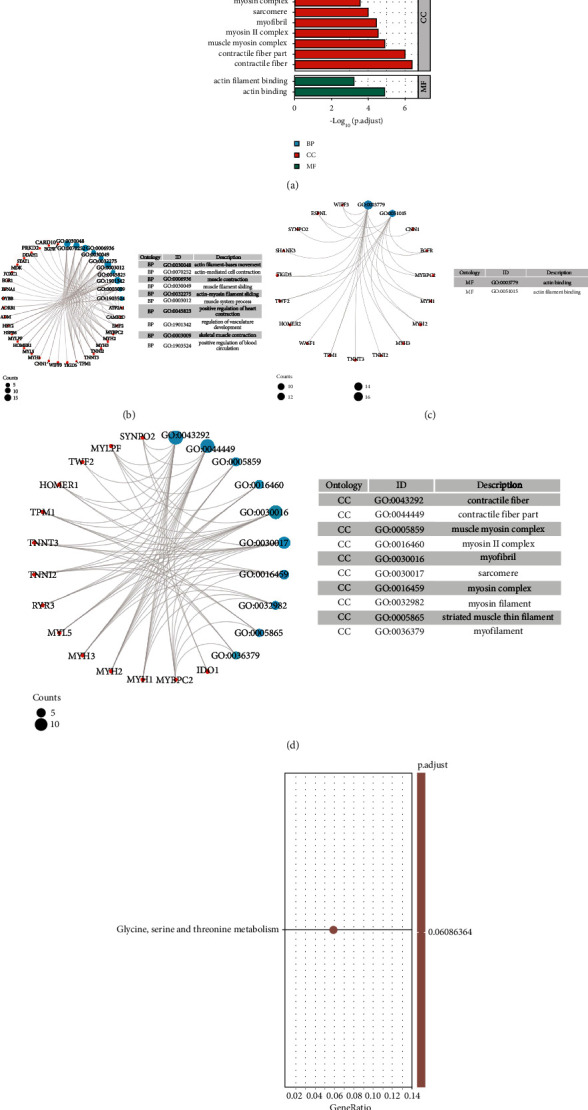
KEGG and GO enrichment analyses of DEGs. (a) The result of the biological process, molecular function, and cellular component-associated GO terms. (b–d) The size of the dot showed count number of enriched genes in the biological process, molecular function, and cellular component. (e) The result of KEGG pathway analysis.

We carried out GO and KEGG enrichment analyses for the exploration of the above differentially expressed genes' potential functions and pathways. Many ontology assignments were found with the use of DAVID software, which included 34 BP, 2 MF, and 12 CC (*p*.adj < 0.05) (Figure 2(a)). According to the BP category, most DEGs played a certain role in the muscle system process, actin-myosin filament sliding, muscle contraction, muscle filament sliding, actin-mediated cell contraction, actin filament-based movement, positive regulation of heart contraction, regulation of vasculature development, positive regulation of blood circulation, and skeletal muscle contraction (Figure 2(b)). According to the MF category, most DEGs were likely to affect actin binding as well as actin filament binding (Figure 2(c)). According to the CC category, most DEGs were found to show a relationship to myosin complex, myosin II complex, muscle myosin complex, contractile fiber part, myofibril, sarcomere, contractile fiber, myosin filament, striated muscle thin filament, and myofilament (Figure 2(d)). Furthermore, as revealed by the KEGG pathway results, the DEGs had significant enrichment within the glycine, serine and threonine metabolism, whereas no significant difference was reported (*p*.adjust = 0.06) (Figure 2(e)).

### 3.4. PPI Network Analysis and Hub Gene Identification

**Figure 3:**
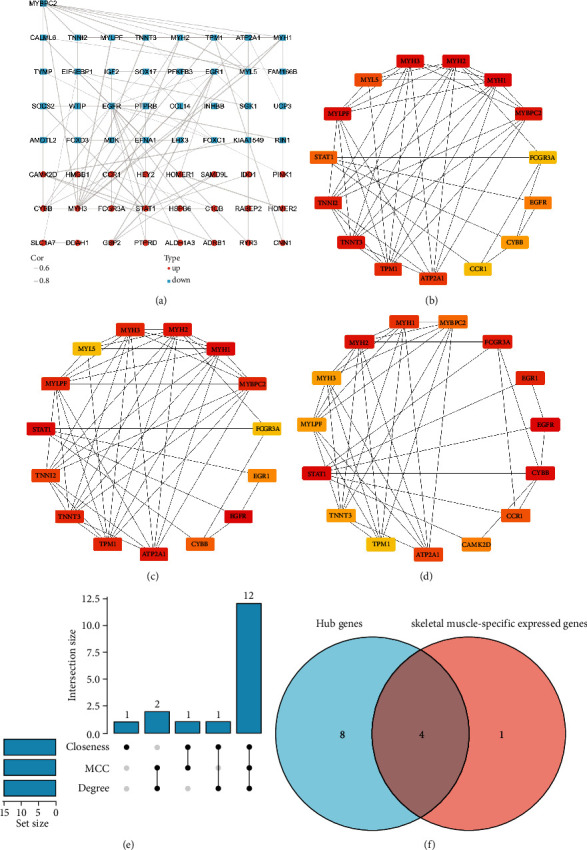
Network analysis of DEGs. (a) Protein-protein interaction network constructed with the differentially expressed genes. Red indicates that gene expression is upregulated; blue indicates that gene expression is downregulated. (b–d) Top 15 genes with the highest MCC, Degree, and Closeness. (e) UpSet diagram summarizing overlapped genes in three sections. (f) Venn diagram of hub genes and skeletal muscle-specific expressed gene overlap.

We built an interaction network of proteins coded by DEGs, covering 106 edges and 57 nodes, on the basis of STRING; Cytoscape was adopted to visualize this network (Figure 3(a)). Subsequently, the CytoHubba plugin was adopted to identify hub genes. Top 15 hub nodes of MCC, Degree, and Closeness subnetworks were retrieved (Figures 3(b)–3(d)), and 12 overlapped genes (MYH2, TPM1, MYBPC2, MYH3, TNNT3, FCGR3A, EGFR, CYBB, STAT1, ATP2A1, MYH1, and MYLPF) were screened out as hub genes (Figure 3(e)) (Table 2). The discovery of genes achieving specific expressions within skeletal muscle of RYR1 mutation-associated myopathies may contribute to the discovery of key targets in the pathogenesis of RYR1 mutation-associated myopathies. Thus, 12 hub genes with genes achieving specific expressions within skeletal muscle were cross-referenced. Lastly, we obtained four hub genes achieving specific expressions within skeletal muscle (MYH1, ATP2A1, TNNT3, and MYLPF) (Figure 3(f)).

### 3.5. ROC Curve of the 4 Hub Genes Achieving Specific Expressions within Skeletal Muscle

**Figure 4:**
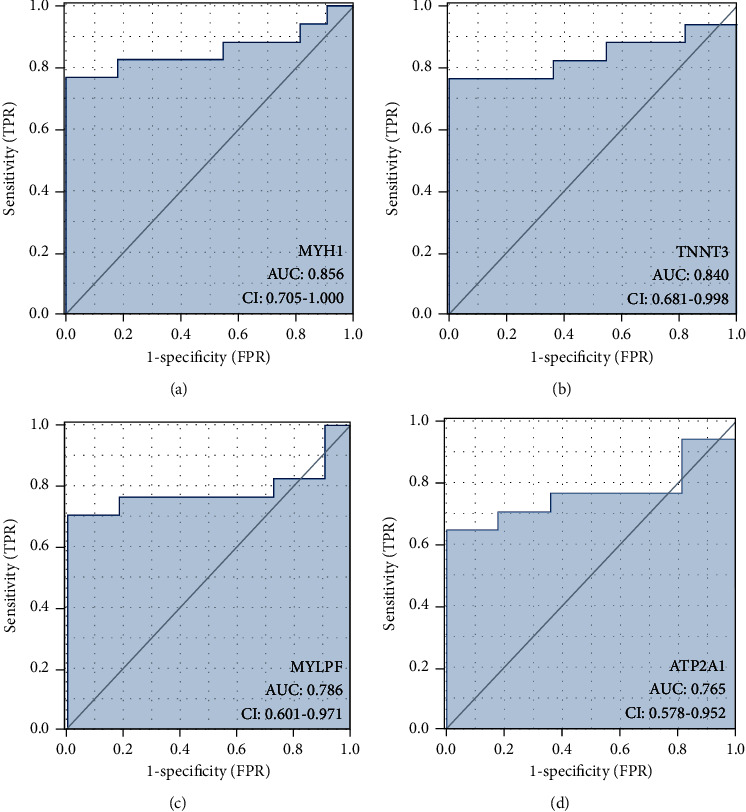
ROC curve of the 4 specifically expressed hub genes. AUC: area under the ROC curve.

Area under the curve (AUC) serves as an indicator integrating specificity and sensitivity, capable of describing diagnostic tests' inherent validity [30]. The above 4 hub genes achieving specific expressions have higher diagnostic value in RYR1 mutation-associated myopathies compared with normal control samples. MYH1 (AUC: 0.856) achieved the maximum diagnostic value in RYR1 mutation-associated myopathies samples (Figure 4(a)), and the diagnostic values of other genes included the following: TNNT3 (AUC: 0.840) (Figure 4(b)), MYLPF (AUC: 0.786) (Figure 4(c)), and ATP2A1 (AUC: 0.765) (Figure 4(d)). Impacted by their good diagnostic properties in RYR1 mutation-associated myopathies, a hypothesis was proposed that MYH1, ATP2A1, TNNT3, and MYLPF are likely to serve as biomarkers for diagnosing RYR1 mutation-associated myopathies.

### 3.6. Transcription Factor Analysis of Hub Genes Achieving Specific Expressions within Skeletal Muscle

**Figure 5:**
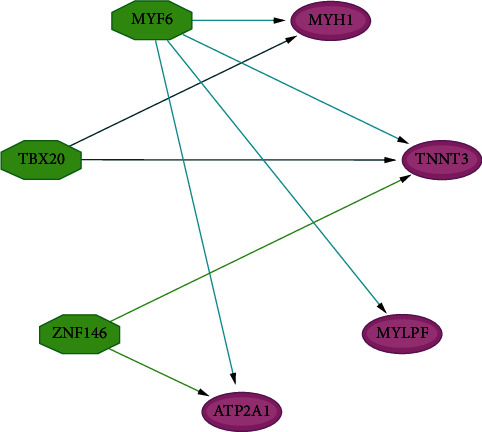
Common TFs among MYH1, ATP2A1, TNNT3, and MYLPF were screened by the iRegulon plugin of Cytoscape software.

Hub genes achieving specific expressions within skeletal muscle transcriptional regulation network are shown in Figure 5. There are three transcription factors with an NES > 5, including MYF6 (NES = 5.751), TBX20 (NES = 5.223), and ZNF146 (NES = 5.151).

## 4. Discussion

RYR1 mutation-associated myopathies is considered the most common nondystrophic muscle disease in humans [31], with an estimated prevalence of 1/400 in an exome analysis from 870 individuals [32]. Diagnosis of RYR1 mutation-associated myopathies is delayed and difficult when there are no clear histopathological features [33]. Moreover, the lack of diagnostic morphological features in muscle biopsy does not exclude the possibility of pathogenic variation in RYR1 [34]. Accordingly, finding suitable biomarkers with diagnostic potential is of great significance for understanding the pathogenesis of RYR1 mutation-associated myopathies and accurate diagnosis as soon as possible.

In this study, 170 DEGs were identified by comparing genes expressed in skeletal muscle samples from healthy and RYR1 mutation-associated myopathies: as revealed by the GO enrichment analysis of all DEGs, actin filament-based movement, actin-mediated cell contraction, muscle contraction, muscle filament sliding, actin-myosin filament sliding, muscle system process, actin binding, myosin II complex, muscle myosin complex, contractile fiber part, contractile fiber, myofibril, sarcomere, and myosin. They were found to be correlated with complex, myosin filament, striated muscle thin filament, etc., which suggested that DEGs are significantly correlated with the function of skeletal muscles.

After obtaining the PPI network screening hub genes, we identified four hub genes achieving specific expressions within skeletal muscle. The ROC curve analysis showed that MYH1, ATP2A1, TNNT3, and MYLPF showed good diagnostic performance for RYR1 mutation-associated myopathies. It is a potential biomarker of RYR1 mutation-associated myopathies with diagnostic efficacy.

There are four myosin heavy chain (MyHC) subtypes in skeletal muscle, including MYH7 (type I), MYH2 (type IIA), MYH1 (type IIX), and MYH4 (type IIB). MYH1 encodes MYH1 (type IIX) in the above four myosin heavy chain (MyHC) subtypes [35]. MYH1 facilitates rapid fiber twitching through glycolysis metabolism while serving as a mediator between MYH2 and MYH4 [35]. Thus far, MYH1 has not been associated with RYR1 mutation-associated myopathies, but it has been found in studies on other muscle diseases that MYH1 mutations in humans and horses are closely related to recurrent rhabdomyolysis [36]. Abnormal expression of MYH1 is closely related to muscle diseases, so MYH1 is of great significance in explaining the pathogenesis, development, and diagnosis of RYR1 mutation-associated myopathies.

Troponin T (TnT) is a core participant in the function of Ca^2+^-regulated actin filaments. It is of great significance in striated muscle contraction. Vertebrate TnT is encoded by three homologous genes, which have specific expressions in slow muscle (TNNT1), cardiac muscle (TNNT2), and fast skeletal muscle (TNNT3) [37]. Abnormal expression of TNNT3 mutations has been reported to cause congenital myopathy (e.g., nemaline myopathy and distal arthrogryposis) [38]. Accordingly, TNNT3 is likely to be critical to the disease progression of various types of congenital myopathy. In addition, TNNT3 was significantly reduced in skeletal muscle of RYR1 mutation-associated myopathies and had a high diagnostic value for RYR1 mutation-associated myopathies (AUC: 0.840). TNNT3 is recognized as a novel effective biomarker for diagnosing RYR1 mutation-associated myopathies.

MYLPF is capable of encoding the regulatory light chain of striated muscle [39]. MYLPF genes are of high importance to fast and slow development of skeletal muscle [40]. A study by Bamshad et al. suggested that partial loss of MYLPF function can lead to congenital contracture, possibly due to skeletal muscle degeneration of the distal limb. MYLPF knockout was used to simulate MYLPF injury in zebrafish, and it was found that MYLPF knockout reduced trunk contractility and complete pectoral fin paralysis, thus revealing that MYLPF injury could have a serious effect on limb motor function [41]. This implied that the normal expression of MYLPF could play a vital role in maintaining the normal function of skeletal muscle. In this study, MYLPF was significantly reduced in skeletal muscle of RYR1 mutation-associated myopathies, which may be critical to explain the development of myopathies related to RYR1 mutation. MYLPF (AUC: 0.786) has good diagnostic efficacy against RYR1 mutation-associated myopathies.

Skeletal muscle contraction and relaxation are adjusted by cytoplasmic calcium concentration. Calcium is first released from the sarcoplasmic reticulum through the Ryanodine receptor 1 (RYR1) channel and then binds to troponin C to induce contraction [42]. Sarcoplasmic/endoplasmic reticulum Ca^2+^-ATPase 1 is a membrane-binding protein that pumps Ca^2+^ from the cytoplasmic matrix to SR and is coupled to ATP utilization [43], which leads to relaxation. There are two major subtypes of SERCA in mammalian cells, of which the main one is SERCA1/ATP2A1, which is expressed in rapid twitch (type 2) skeletal muscle fibers [44]. DNA changes at ATP2A1 loci (e.g., whole gene deletions, single exon deletions, splice site mutations, and small exon transposition deletions or insertions), and missense substitutions can result in Brody myopathy, which is an autosomal recessive myopathy [44–46]. The clinical manifestations, genetic patterns, and prevalence of myopathies vary in Brody myopathy and RYR1 mutation-associated myopathies, whereas an exon group analysis of a malignant hyperthermic family reported that all members of the family had ATP2A1 deletion of Brody myopathy. Malignant hyperthermia susceptibility was also significantly correlated with RYR1 mutations, so whether ATP2A1 is correlated with RYR1 is worth discussing. The limited proteolysis of RYR1, a member of the Brody family, revealed that the RYR1 protein in the Brody family was specific and might arise from an unidentified mutation of ATP2A1 [44]. Proteolysis of RYR1 may occur after tissue harvesting; no significant impairment of excitation-contraction coupling was reported in the patients studied, which implies that the protein remained fully functional throughout its life.

There are some limitations in the present research. First, the sample sizes of the dataset used in this study was small, so the results obtained from the perspective of bioinformatics should be further verified. The expanded number of samples should be studied to increase the credibility of results. Second, most of our data were obtained from specimens of RYR1 mutation-associated myopathy patients and normal healthy subjects through the biological information assay. Further experimental analysis is still required to validate the above findings.

## 5. Conclusion

The results of this study contribute to the explanation of the pathogenesis of RYR1 mutation-associated myopathies and are beneficial to find out the molecular mechanism of the pathological process. This study provides a reference for the diagnosis and therapeutic targets of RYR1 mutation-associated myopathies and helps anesthesiologists choose a reasonable anesthesia mode and prepare for malignant hyperthermia in patients.

## Figures and Tables

**Table 1:** Distribution of tissue-/organ-specific expressed gene.

System/organ	Genes	Counts
Skeletal muscle	MYH1, ATP2A1, TNNT3, MYLPF, TNNI2	5
Pineal	GDF10, DDIT4L, PPP1R1C, LHX3, AMOTL2	5
Placenta	TPPP3, EGFR, HOMER2, ADM	4
Adipocyte	WISP2, MGST1, CRLF1, AQP7	4
Whole blood	FCGR3A, FAM129A	2
Adrenal	DHCR24	1
CD34+	CDCA7	1
Fetal brain	LHX6	1
Burkitt's lymphoma (Raji)	PSPH	1
Liver	ACOX2	1
Lung	EMP2	1
Skin	SCGB1D2	1
Smooth muscle	ELTD1	1
Thyroid	ID4	1
Uterus	HOXA10	1

**Table 2:** The hub genes associated with RYR1 mutation-associated myopathies.

Gene symbol	Adjusted *p* value	Log FC	Regulation
MYH2	0.045127924	-3.406597997	Down
TPM1	0.008090907	-3.691531167	Down
MYBPC2	0.010949967	-4.479919922	Down
MYH3	0.017635365	1.414275217	Up
TNNT3	0.014401027	-3.249590136	Down
FCGR3A	0.026211778	1.41009252	Up
EGFR	0.000498769	-1.235865382	Down
CYBB	0.001106175	1.424261759	Up
STAT1	0.041317013	1.408738498	Up
ATP2A1	0.020403562	-4.045132724	Down
MYH1	0.009859039	-4.370268882	Down
MYLPF	0.040256081	-2.916819502	Down

## Data Availability

The datasets used and/or analyzed during the current study are available from the corresponding author and GEO database (https://www.ncbi.nlm.nih.gov/geo).

## Abbreviations

DEGs: Genes with differential expression
RYR1: Ryanodine receptor 1
GO: Gene Ontology
KEGG: Kyoto Encyclopedia of Genes and Genomes
AUC: Area under the curve
ROC: Receiver operating characteristic
GEO: Gene expression synthesis
MH: Malignant hyperthermia
ETCO2: End-expiratory carbon dioxide
MHS: Malignant hyperthermia susceptibility
CCD: Central core disease
CNM: Central nuclear myopathy
MmD: Multi-micronucleus disease
PPI: Protein-protein interaction
MyHC: Myosin heavy chain
TnT: Troponin T.
